# A tool for screening potentially inappropriate prescribing in Chinese children

**DOI:** 10.3389/fphar.2022.1019795

**Published:** 2022-10-31

**Authors:** Siyu Li, Liang Huang, Linan Zeng, Dan Yu, Zhi-Jun Jia, Guo Cheng, Lingli Zhang

**Affiliations:** ^1^ Department of Pharmacy, West China Second University Hospital, Sichuan University, Chengdu, China; ^2^ Evidence-bBsed Pharmacy Center, West China Second University Hospital, Sichuan University, Chengdu, China; ^3^ NMPA Key Laboratory for Technical Research on Drug Products In Vitro and In Vivo Correlation, Chengdu, China; ^4^ Key Laboratory of Birth Defects and Related Diseases of Women and Children, Ministry of Education, Chengdu, China; ^5^ West China School of Medicine, Sichuan University, Chengdu, China; ^6^ Department of Pediatrics, West China Second University Hospital, Sichuan University, Chengdu, China; ^7^ West China School of Pharmacy, Sichuan University, Chengdu, China; ^8^ Laboratory of Molecular Translational Medicine, Center for Translational Medicine, Sichuan University, Chengdu, China

**Keywords:** children, inappropriate prescribing, inappropriate prescription, potentially inappropriate medication, prescribing omission, screening tool

## Abstract

**Background:** More than half of adverse drug events in pediatric patients are avoidable and blocking medication errors at the prescribing stage might be one of the most effective preventive measures.

**Objective** **:** To form a tool (a series of criteria) for detecting potentially inappropriate prescriptions in children, promote clinical rational drug use and reduce risks of medication in children.

**Methods:** Potentially inappropriate prescription propositions for children were collected through a systematic review. Then, the Delphi technique was adopted to form the final criteria. Panelists were asked to use a 5-point Likert scale to rate their agreement with each potentially inappropriate prescription proposition and were encouraged to add new propositions based on their clinical experience and knowledge. After 2 rounds of Delphi survey and propositions were fully revised and improved, the final criteria for identifying potentially inappropriate prescriptions in children were formed.

**Results:** The final criteria for identifying potential inappropriate prescriptions in children has 136 propositions, which were divided into “criteria for children with non-specific diseases/conditions” (71 propositions: 68 for potentially inappropriate medication, 3 for potential prescribing omission) and “criteria for children with specific diseases/conditions” (65 propositions: 55 for potentially inappropriate medication, 10 for potential prescribing omission), according to whether the proposition was about identifying specific risks associated with one drug in children with a specific other diseases/conditions that do not exist in children with other diseases/conditions.

**Conclusion:** A tool for screening potentially inappropriate prescriptions in children is formed to detect potentially inappropriate medication and prescribing omission in pediatrics and is available to all medical professionals liable to prescribe or dispense medicines to children.

## 1 Introduction

Prescriptions are generally considered appropriate when medicines on prescriptions have a clear evidence-based indication, are well tolerated in the majority of patients and are cost-effective. Conversely, prescriptions that do not meet the above conditions are considered inappropriate prescriptions. Potentially inappropriate prescriptions (PIPs) are prescriptions with potential risks that outweigh the benefits, which are more likely to be ultimately determined to be inappropriate than other prescriptions after a thorough review by clinicians or pharmacists. PIP consists of two parts—potentially inappropriate medication (PIM) and potential prescribing omission (PPO). PIM is medication when the potential risks of adverse drug events outweigh the potential clinical benefits, especially when safer or more effective alternatives are available. The inappropriateness of PIM mainly includes prescribing errors (inappropriate medicine selection, dosage, duration, drug-disease interaction, drug-drug interaction or drug-food interaction, etc.) and overprescribing. PPO is the omission of prescribing medicines with significant benefits to the patient’s length or quality of life in the absence of contraindications, underprescribing of beneficial drugs (Gallagher et al., 2008a).

Over the past three decades, many implicit or explicit indicators or criteria were developed to help detect PIPs in the ederly (Kaufmann et al., 2014; O'Connor et al., 2012; Spinewine et al., 2007). (an indicators or criteria is considered explicit if it consists of a series of clear and specific propositions.) Compared with implicit indicators (e.g., medication appropriateness index (MAI) (Hanlon et al., 1992; Hanlon and Schmader, 2013)), explicit criteria (e.g., START/STOPP criteria (O'Mahony et al., 2015) and Beer criteria (By the 2019 American Geriatrics Society Beers Criteria® Update Expert Panel, 2019)) require less clinical knowledge and experience of users, and are easier to implement manual or automated prescription review (Gillespie et al., 2013; Huibers et al., 2019a). It has been found in the elderly population that some explicit PIP criteria (such as START/STOPP criteria) have good applicability and reliability (Gallagher et al., 2008b; Gallagher et al., 2011; O'Mahony, 2020), and their use in PIPs screening can significantly improve the rationality of drug use, reduce adverse drug reactions (ADRs), readmissions, falls and medicine costs in elderly patients (Hill-Taylor et al., 2016).

As the other special population, children’s prescription quality has always been the focus of many medical workers and researchers. Due to the difficulty in conducting clinical trials in children (difficulties in recruiting and organizing subjects), information on children’s medication is lacking and the safety, efficacy and economic interests of many drugs in children are unknown. A large number of off-label and high-risk pediatric prescriptions cause serious safe problems and greatly increase medication risk in children (from mild rashes to serious adverse reactions such as preventable death and prolonged hospitalization) (Balan et al., 2018; Shuib et al., 2021). In the pediatric population, the incidence of ADRs in inpatients is 9.53% and in outpatients is 1.46%; and the incidence of ADRs leading to hospital admission in children is 2.09% (Impicciatore et al., 2001). Unlike the elderly, the development of explicit PIP criteria for children is in its infancy, and there are only five PIP criteria for children (Prot-Labarthe et al., 2011; Barry et al., 2016; Corrick et al., 2019; Meyers et al., 2020; Sadozai et al., 2020; Li et al., 2022). Experts in France were the first to develop the PIP criteria for children. They released the POPI criteria (Pediatrics: Omission of Prescriptions and Inappropriate Prescriptions) in 2011 (Prot-Labarthe et al., 2011), then the United Kingdom (Barry et al., 2016; Corrick et al., 2019) and the US (Meyers et al., 2020) successively released their PIP criteria for children. We previously conducted a comprehensive systematic review on existing tools for identifying PIPs in children and their applicability in clinical practices (Li et al., 2022) and regrettably found that China has not yet developed a tool for detecting PIPs in children based on its actual clinical practice.

It is reported that children accounts for about 30% of the total population and pediatric diseases account for about 20% of all medical consultations in China. The incidence of ADRs in children in China is twice that in adults (in neonates is four times); About 7 000 children die of medication errors every year and the incidence of irrational drug use is 12%–32% (Tang, 2014; National Medical Products Administration, 2020; Cui et al., 2021; Zhao, 2021); Among children under the age of 14, approximately 30,000 children are deaf each year due to inappropriate medication (Zhao, 2021). Our study aimed to form a series of criteria for detecting PIPs in children, with a view to applying them to review and intervene in pediatric prescribing, to promote clinical rational drug use and reduce medication risk in children.

## 2 Materials and methods

### 2.1 Forming preliminary children’s potentially inappropriate prescription criteria

#### 2.1.1 Systematically searching and extracting children’s potentially inappropriate prescription propositions

Medline (Ovid), Embase (Ovid), Cochrane Library, CNKI, VIP, and Wanfang Data were systematically searched to identify articles related to children’s PIP. Moreover, reference lists of included articles, children’s medication information from national or provincial “Key Drug List for Monitoring Rational Drug Use,” “Adverse Drug Reaction Monitoring Report” and “Drug Alert Report” were used as supplementary search sources to identify additional children’s PIP propositions. The retrieval time of databases was as of July 2021. Then, we reviewed all articles related to PIP in children (age <18 years) and extracted PIP criteria or propositions from them. The detailed search strategy, eligibility criteria and literature screening and selection results could be found in Supplementary Material S1.

#### 2.1.2 Searching evidence related to included potentially inappropriate prescription propositions

For each PIP proposition, we searched for relevant evidence including clinical guidelines, systematic reviews, original clinical studies, expert consensus, National Children’s Formulary, drug package inserts and other supplemental materials provided by pharmaceutical companies, etc. The preliminary children’s PIP criteria were formed after removing PIP propositions that were not supported by children’s evidence or without children’s evidence.

### 2.2 Delphi method

We revised and validated the preliminary criteria by a two-round modified Delphi (Hasson et al., 2000) before finally forming the children’s PIP criteria. The purpose of conducting Delphi method is to achieve a convergence of opinion and a general consensus on a particular topic, by questioning experts through successive questionnaires.

#### 2.2.1 Selection of the Delphi panel

The criteria for selecting the members of the expert panel were as follows: 1) Clinicians or pharmacists; 2) Deputy senior professional title or above; 3) Engaged in pediatric clinical work ≥10 years; 4) The workplace of the expert is a tertiary hospital 5) Interested in our study and able to complete 2 rounds of questionnaires on time.

The reason why we chose clinicians or pharmacists from tertiary hospitals and engaged in pediatric clinical work ≥10 years as panelists was that experts from tertiary hospitals might have better access to the latest and best evidence and knowledge, more experience with medication, and more diverse and complex patients than those from primary health care and those engaged in pediatric clinical practice for a short time in China.

#### 2.2.2 Data collection and analysis

**FIGURE 1:**
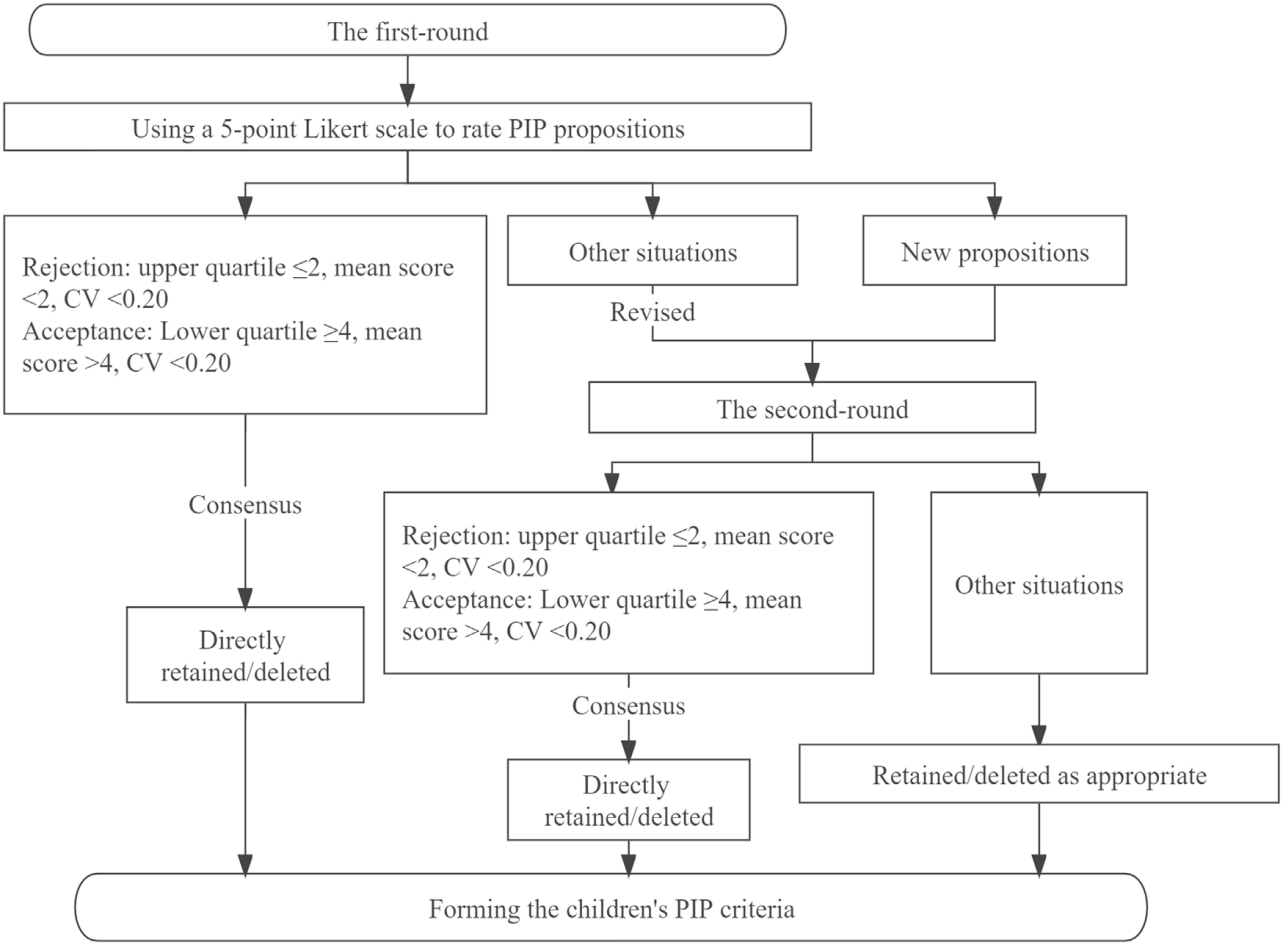
Delphi survey process.

Electronic questionnaires were sent to the panelists by e-mail. The panelists were asked to comprehensively evaluate the clinical applicability and feasibility of the preliminary children’s PIP criteria, and use a 5-point Likert scale to rate their agreement for each PIP proposition. One point and 5 points respectively meant “completely disagree” and “completely agree”, and for propositions rated < 4 points, panelists were required to provide reasons why the proposition was unreasonable or unfeasible. Panelists could comment on existing PIP propositions or propose new PIP propositions based on their clinical experience, while encouraging them to cite appropriate evidence to support these new PIP propositions. Each of the panelists who had participated in the first round was sent the second-round questionnaire with feedback on the results of the first round (including the average panel rating and the full score rate). The panelists were then asked to re-rate revised propositions without consensus based on both their opinion and the group response to the previous round (Figure 1).

Criteria for reaching consensus were set before starting the Delphi survey. When the upper quartile ≤2 points, the mean score < 2 points and the coefficient of variation (CV) < 0.20, this indicated there was consensus by the Delphi panel members on rejection of the PIP proposition. When the lower quartile ≥4, the mean score > 4 points, CV < 0.20, this indicated there was consensus by the Delphi panel members on acceptance of the PIP proposition. Other situations indicated a lack of consensus among experts, and after revision, these propositions would along with new propositions proposed by panelists enter the second round. If consensus was not reached after the second round, the proposition was retained or deleted as appropriate based on the principles of scientificity and feasibility, and expert comments.

The calculation of the mean score ( $\bar{X}$ ), *CV*, median score (*M*), interquartile range (*IQR*) and full score rate (proportion of experts who gave 5 points) of each PIP proposition was performed by Excel 2019.

## 3 Results

### 3.1 Preliminary children’s potentially inappropriate prescription criteria

**FIGURE 2:**
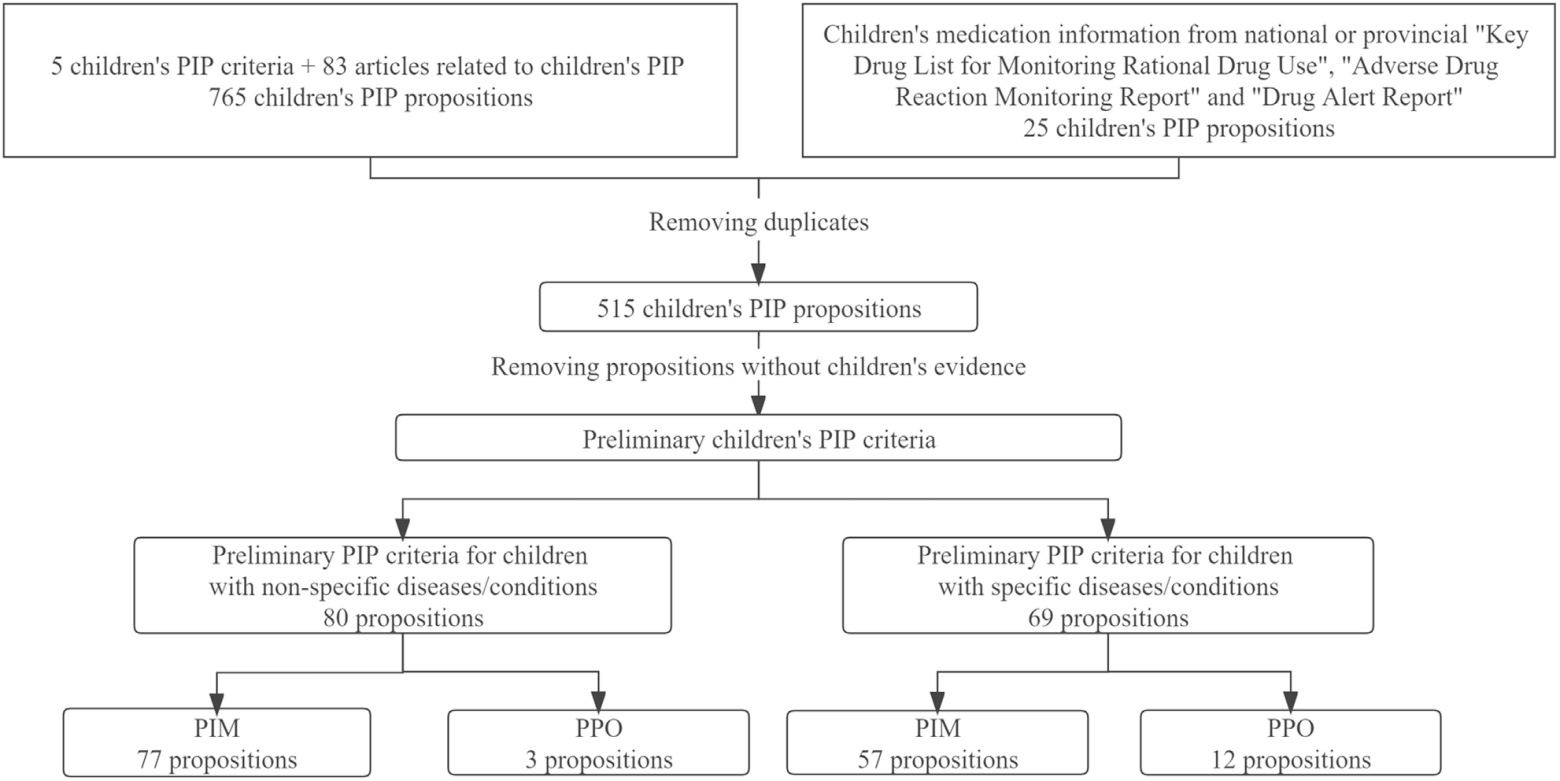
Formation process of the preliminary children’s PIP criteria.

A total of 787 propositions for children’s PIP were extracted, and 515 propositions were retained after removing duplicates. A total of 366 propositions without children’s evidence were excluded, then the preliminary children’s PIP criteria (149 propositions) were formed. According to whether the proposition could only be used to detect PIPs in children with specific diseases or conditions, propositions were divided into two parts—“PIP criteria for children with non-specific diseases/conditions” [e.g., “Tricyclic antidepressants desipramine and imipramine in children (PIM),” “Chloramphenicol in neonates” (PIM)”] and “PIP criteria for children with specific diseases/conditions” [e.g., “Codeine for children after tonsillectomy and adenoidectomy (PIM)”, “Loperamide for children <4 years or with acute infectious diarrhea (PIM)”, “Oral rehydration solution (ORS) for dehydrated children unless intravenous fluid therapy is indicated (shock, red flag symptoms despite ORS, persistent vomiting of ORS) (PPO)”]. Finally, there were a total of 80 preliminary PIP propositions in “PIP criteria for children with non-specific diseases/conditions”, including 77 PIM propositions and 3 PPO propositions; the “PIP criteria for children with specific diseases/conditions” consisted of 69 preliminary PIP propositions, including 57 PIM propositions and 12 PPO propositions (Figure 2).

### 3.2 Composition of the Delphi panel

In total, 19 pediatric specialists from tertiary hospitals were invited to participate in a Delphi panel to develop these criteria. In the end, A total of 16 specialists agreed to participate. The panel consisted of 11 clinicians and 5 pharmacists and they specialized in pediatric emergency and critical care, pediatric infection, pediatric hematology, pediatric gastroenterology, pediatric rheumatology, pediatric respiratory, pediatric neurology, and clinical pharmacy. Moreover, 11 specialists (69%) engaged in pediatric clinical work for more than 20 years.

### 3.3 Children’s potentially inappropriate prescription criteria

**FIGURE 3:**
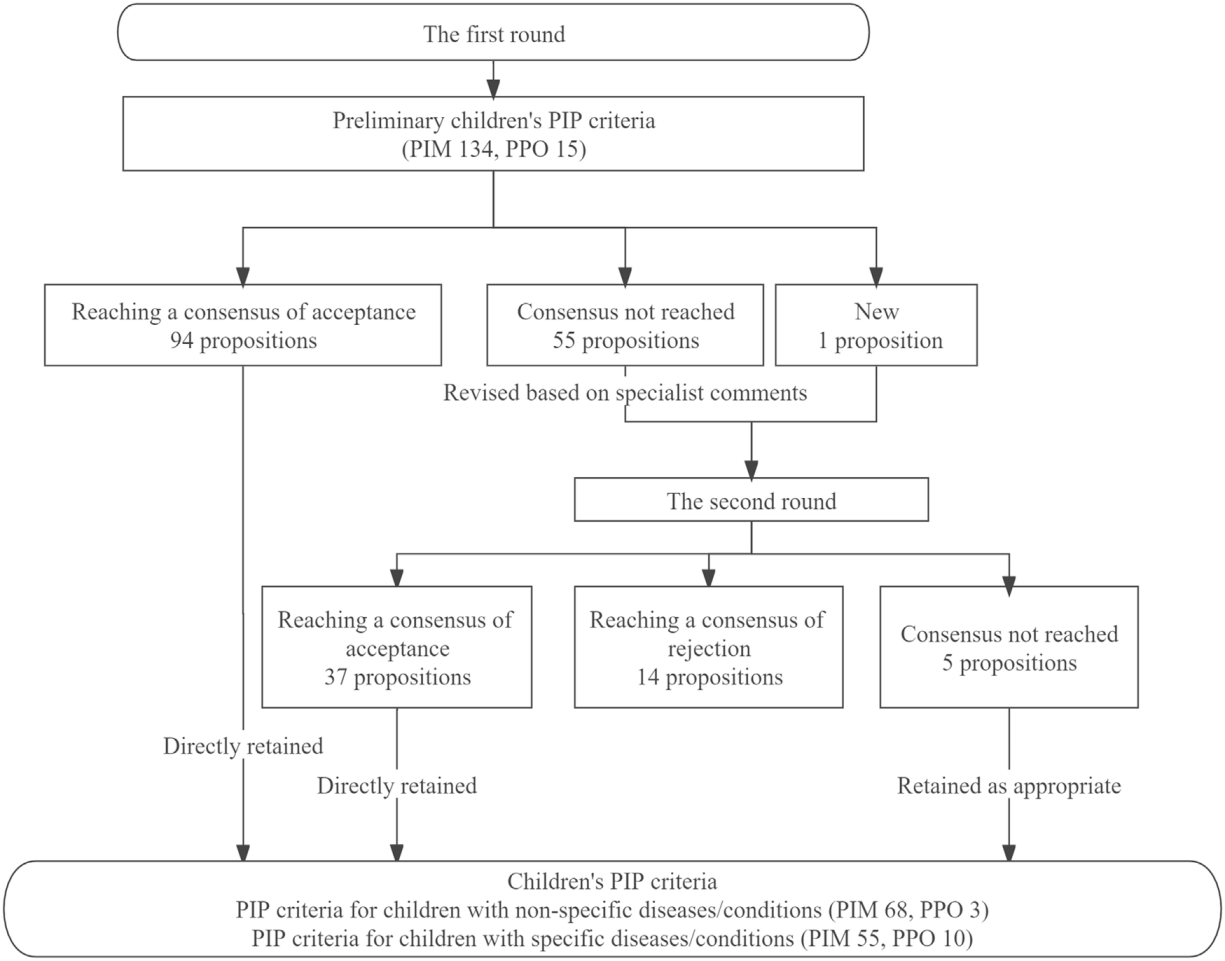
Results of the Delphi survey.

After the first-round Delphi survey, 94 propositions reached a consensus and were directly retained, and none of the propositions was directly rejected. Fifty-five propositions without consensus were revised according to specialists’ comments and entered into the second round together with 1 new proposition proposed by specialists in the first round, and 42 propositions were finally retained after reviewing specialists’ comments and suggestions. Figure 3 presented the results of the Delphi survey.

A total of 136 PIP propositions were included in the final children’s PIP criteria, 71 propositions for PIP criteria in children with non-specific diseases/conditions (PIM 68, PPO 3), and 65 propositions for PIP criteria in children with specific diseases/conditions (PIM 55, PPO 10).

The PIP criteria in children with non-specific diseases/conditions included anti-infective drugs, nervous system drugs, Chinese patent medicines, digestive system drugs, respiratory system drugs, dermatological drugs, anti-tumor drugs and immune drugs, sensory organs drugs, cardiovascular system drugs, skeletal-muscular system drugs, antiparasitic drugs. The numbers of PIP propositions for anti-infective drugs and nervous system drugs were the two largest, with 22 and 15 propositions, respectively (Table 1).

**TABLE 1:** PIP criteria for children with non-specific diseases/conditions.

PIM/PPO	Medicine	Potentially inappropriate prescription	Risk/Recommendation
**Nervous system**			
PIM	Propofol	Propofol doses >4 mg/kg/h for more than 48 h in children	Risk of propofol-related infusion syndrome, higher rate in children than adults because higher relative doses of propofol are needed, especially in status epilepticus. Avoid doses >4 mg/kg/h over 48 h in children
PIM	Dopamine antagonists (chlorpromazine, fluphenazine, haloperidol, droperidol, perphenazine, trifluoperazine, etc.)	Dopamine antagonists in children, especially in <2 years	Risk of acute dystonia (dyskinesia); Intravenous use will increase the risk of respiratory depression, extravasation, and death. Avoid in <2 years; Caution in children
PIM	Opioids (morphine, tramadol, pethidine, codeine, dihydrocodeine, sufentanil)	Opioids in children, especially in <2 years	Risk of respiratory depression. Avoid in <2 years (neonates, especially premature neonates are at the highest risk); Caution in children
PIM	Naloxone	Naloxone in neonates or children with known or suspected opioid physical dependence	Risk of seizures. Avoid in neonates; Caution in children with known or suspected opioid physical dependence, including neonates whose mothers are opioid dependents
PIM	Benzocaine	Benzocaine in <2 years	Risk of methemoglobinemia. Avoid in <2 years for teething or pharyngitis
PIM	Lidocaine	Lidocaine in <6 years	Risk of seizures, arrhythmia, and death (due to central nervous system depression, seizures, or arrhythmia). Avoid in <2 years for teething pain (lidocaine 2% viscous); Caution in local anesthesia in children <6 years
PIM	Atypical/second-generation antipsychotics (clozapine, risperidone, olanzapine, quetiapine, ziprasidone, aripiprazole, perospirone, paliperidone, amisulpride, etc.)	Atypical antipsychotics (especially clozapine, risperidone, quetiapine) in children for more than 24 weeks	Risk of agranulocytosis (especially clozapine, risperidone, quetiapine) and abnormal glucose and lipid metabolism (increasing blood lipids, weight gain, elevating blood glucose even causing diabetes). Caution in children; Avoid long-term use (>24 weeks)
PIM	Diazepam	Diazepam in <2 years, especially in neonates	Risk of sedation. Avoid in neonates; Caution in <2 years
PIM	Midazolam	Midazolam in neonates with very low birth weight (<1500 g)	Risk of severe intraventricular hemorrhage, periventricular leukomalacia, and death. Avoid in neonates with very low birth weight (<1500 g)
PIM	Phenytoin	Phenytoin in <1 year or children not undergoing TDM	Neurotoxicity, special pharmacokinetics, and symptoms of poisoning are difficult to identify. Avoid in <1 year; Caution in children not undergoing TDM (It is recommended for children to carry out TDM)
PIM	Tricyclic antidepressants	Desipramine or imipramine in children	Risk of sudden cardiac death. Avoid in children (desipramine); Caution in children (imipramine)
PIM	SSRIs and SNRIs	SSRIs or SNRIs (especially paroxetine and venlafaxine) in children	Increasing suicide risk. Avoided in children (paroxetine and venlafaxine); Caution in children (other SSRIs and SNRIs). It is recommended that children using SSRIs or SNRIs be closely monitored, especially during the first few months or when changing the dose or treatment regimen
PIM	Valproic acid and its derivatives	Valproic acid and its derivatives in <2 years, or in children with metabolic or mitochondrial diseases or taking other antiepileptic drugs such as phenytoin	Risk of pancreatitis and fatal hepatotoxicity. Avoid in <2 years, especially in children with metabolic or mitochondrial diseases, or are taking other antiepileptic drugs such as phenytoin
PIM	Lamotrigine	Lamotrigine in <2 years	Risk of serious skin rash. Caution in children; TDM is recommended during medication
PIM	Antiepileptic drugs (AEDs)	Antiepileptic drugs, especially AEDs with liver enzyme induction (such as phenytoin, carbamazepine, oxcarbazepine), in children	Risk of osteoporosis, long-term use will reduce bone density; Risk of severe rash (carbamazepine), such as severe erythema multiforme type drug eruption, epidermolysis bullosa type drug eruption and exfoliative dermatitis, which occurs more frequently in children >6 years. Caution in children
**Antiinfectives For Systemic Use**			
PIM	Indinavir	Indinavir in children	Risk of nephrolithiasis. Avoid in children
			Risk of hyperbilirubinemia. Avoid in neonates
PIM	Atazanavir	Atazanavir in neonates	Risk of kernicterus. Caution in neonates, unless pharmacogenetic testing is implemented
PIM	Darunavir	Darunavir in <3 years or <10 kg	Risk of seizures and death. Avoid in <3 years or <10 kg
PIM	Chloramphenicol	Chloramphenicol in neonates	Risk of gray baby syndrome, irreversible bone marrow suppression, and aplastic anemia. Avoid neonates, unless the blood concentration is monitored
PIM	Cefathiamidine	Cefathiamidine (injection) is once a day instead of 2–4 times as specified in the drug package insert	Higher risk of severe allergic reactions such as anaphylactic shock. Avoid once a day, which increases the risk of adverse reactions, and fails to maintain effective blood drug concentrations and produce therapeutic effects
PIM	Ceftriaxone	Ceftriaxone in neonates, especially in premature neonates	Risk of hyperbilirubinemia, neonates (especially premature neonates) may develop kernicterus. Caution in neonates
PIM	Azithromycin and erythromycin (oral or intravenous)	Azithromycin or erythromycin (oral or intravenous) in neonates ≤14 days	Risk of hypertrophic pyloric stenosis. Avoid in neonates ≤14 days, unless treating *Bordetella* pertussis (azithromycin), or *Chlamydia trachomatis* pneumonia (azithromycin and erythromycin)
PIM	Lincomycin	Lincomycin in children	Risk of neuromuscular blockade (dyspnea, weakness, dysphagia), shock-like reaction, epidermolysis bullosa, and hearing loss (more common in children). Caution in children
PIM	Aminoglycoside antibiotics (amikacin, streptomycin, gentamicin, etimicin, etc.)	Aminoglycoside antibiotics in <6 years, except for children with drug-resistant *tuberculosis* or undergoing TDM	Risk of ototoxicity and nephrotoxicity. Avoid in <6 years, except for children with drug-resistant *tuberculosis* or undergoing TDM
PIM	Voriconazole	Voriconazole in children	Risk of hepatotoxicity, pancreatitis, and skeletal fluorosis. Caution in children
PIM	Cefradine	Cefradine in children	Risk of nephrotoxicity. Caution in children
PIM	Nitrofuran antibiotics	Nitrofuran antibiotics in children	Risk of nephrotoxicity, peripheral neurotoxicity, interstitial pneumonia. Avoid in neonates; Caution in children
PIM	Tetracycline	Tetracycline in <8 years	Risk of tooth discoloration, enamel hypoplasia, and retardation of skeletal development and bone growth in premature neonates. Caution in <8 years
PIM	Sulfonamides (silver sulfadiazine, sulfadiazine, sulfamethoxazole)	Sulfonamides are used in neonates (especially premature neonates), except as an adjuvant treatment for congenital toxoplasmosis (sulfadiazine)	Risk of kernicterus and hemolytic anemia. Avoid in neonates, especially premature neonates, except as an adjuvant treatment for congenital toxoplasmosis (sulfadiazine)
PIM	Quinolones (levofloxacin, ciprofloxacin, ofloxacin, etc.)	Quinolones in children	Bone and cartilage toxicity. Caution in children
PIM	Ceftriaxone	Intravenous calcium-containing preparations within 48 h after ceftriaxone in children	Risk of formation of ceftriaxone-calcium salt deposits. Avoid concomitant use of ceftriaxone with calcium-containing preparations (most total parenteral nutrition (TPN) formulas for children contain calcium salts), and do not inject calcium-containing medicines within 48 h of using ceftriaxone)
PIM	Fluoroquinolones (levofloxacin, ciprofloxacin, ofloxacin, etc.)	Fluoroquinolones and compounds containing divalent or trivalent cations (DTCC) are simultaneously used in children; Taking levofloxacin <2 h before or after taking DTCC; Taking ciprofloxacin <2 h before taking DTCC or <6 h after taking DTCC	Intestinal fluoroquinolones (FQ) and DTCC (including aluminum, magnesium, calcium, iron, or zinc) used at the same time can reduce absorption and bioavailability of FQ, which can lead to treatment failure. Avoid simultaneous use. It is recommended to take levofloxacin at least 2 h before or after DTCC and ciprofloxacin at least 2 h before or 6 h after DTCC
PIM	Aminoglycosides and first-generation cephalosporins	Aminoglycosides and first-generation cephalosporins such as cefradine are simultaneously used in children	Combined use can increase risk of nephrotoxicity and hematuria. Avoid simultaneous use in children
PIM	Vancomycin	An intravenous bolus of vancomycin in children	Risk of severe hypotension and upper body flushing, even cardiac arrest and shock. Avoid intravenous bolus of vancomycin in children. Slow infusion (>1 h) with appropriate dilution is recommended
PPO	Penicillin antibiotics	A skin test should be prescribed to children using penicillin antibiotics unless drug package inserts indicating that the skin test is not necessary before use	Risk of severe allergic reactions such as anaphylactic shock. A skin test must be performed before use, including patients who have ever used penicillin and were not allergic
PPO	Vancomycin	TDM should be implemented in children using vancomycin	Conducting TDM in children using vancomycin could improve clinical efficacy and avoid adverse reactions. Adverse reactions are more likely to occur when the peak concentration >80 μg/ml or the trough concentration >20 μg/ml (trough concentration >10 μg/ml in neonates) in children
PPO	Aminoglycosides (amikacin, streptomycin, gentamicin, etimicin, etc*.*)	TDM should be implemented in neonates with gestational age <32 weeks or very low birth weight (<1500 g) and children with cystic fibrosis when using aminoglycosides	Neonates with gestational age <32 weeks or very low birth weight (<1500 g) and children with cystic fibrosis should routinely undergo TDM. For children with good renal function, TDM can be considered, but is not routinely used
**Respiratory System**			
PIM	Carbocysteine	Carbocysteine in children, especially in <2 years	No evidence of its effectiveness in children and its safety in children <2 years is unknown. Avoid in children, especially in <2 years
PIM	Sedative antihistamines (diphenhydramine, promethazine, chlorpheniramine, etc.)	Sedative antihistamines in <2 years	Potential risks of life-threatening side effects, such as respiratory depression. Avoid in <2 years
PIM	Aminophylline	Aminophylline in children with doses >10 mg/kg/d or in neonates	Higher risks of convulsions, arrhythmia, severe hypotension, and cardiac arrest in children with doses >10 mg/kg/d or in neonates. Avoid in children with doses >10 mg/kg/d and neonates, and 5–6 mg/kg/d for children is recommended
PIM	Fluticasone propionate	Long-term high-dose (>500 μg/d) fluticasone propionate in <16 years	Risk of growth delay and slow weight gain. Avoid long-term high-dose (>500 μg/d) use in <16 years, and doses ≤200 μg/d is recommended
PIM	Beclomethasone (nasal use)	Nasal use of beclomethasone in <6 years	Risk of suppression on children’s growth and the hypothalamic-pituitary-adrenal axis function. Compared with other intranasal corticosteroids, beclomethasone has a higher absorption rate. Avoid in <6 years
PIM	Naphazoline hydrochloride	Naphazoline hydrochloride in <2 years	High risk of poisoning. Caution in <2 years
**Alimentary Tract And Metabolism**			
PIM	Sodium/calcium polystyrene sulfonate	Sodium/calcium polystyrene sulfonate in children with very low birth weight (<1500 g)	Risk of colon perforation. Avoid in children with very low birth weight (<1500 g)
PIM	Metoclopramide	Metoclopramide in children, especially in <2 years	Risk of acute dystonia/dyskinesia, increased risk of respiratory depression, extravasation, and death with intravenous use. Avoid in <2 years; Caution in children
PIM	Compound diphenoxylate [diphenoxylate-atropine, (Lomotil)]	Compound diphenoxylate in <2 years	Risk of respiratory depression and death. Avoid in <2 years
PIM	Sodium phosphate solution (rectal enema)	Sodium phosphate solution (rectal enema) in <2 years	Risk of electrolyte abnormalities, acute kidney injury, arrhythmia, and death. Avoid in <2 years
PIM	Lipoic acid	Lipoic acid in <2 years or its doses >30 mg/kg in children	Risk of refractory convulsions. Caution in children with doses >30 mg/kg and in <2 years
PIM	Domperidone and erythromycin	Domperidone and erythromycin are simultaneously used in children	Erythromycin inhibits the metabolism of domperidone and the blood concentration of domperidone can be increased up to 3 times, which increases risk of QT interval prolongation. Avoid simultaneous use in children
**Cardiovascular System**			
PIM	Verapamil	Verapamil in <1 year	Risk of cardiac arrest. Avoid in <1 year
PIM	Camphor	Camphor in children	Risk of seizures. Caution in children
**Musculo-skeletal System**			
PIM	Zoledronic acid	Zoledronic acid in children	Risk of flu-like symptoms, hypocalcemia, and hypophosphatemia. Caution in children
**Dermatologicals**			
PIM	Topical corticosteroids (medium, high, and very high titers, such as clobetasol, betamethasone, etc.)	Topical corticosteroids in <1 year (e.g., for the treatment of diaper dermatitis) or use for more than 2 months in children	Higher risk of hypothalamic-pituitary-adrenal axis inhibition, because the absorption rate in children is higher than adults. Avoid in <1 year and use for more than 2 months
PIM	Isotretinoin	Oral isotretinoin in children <12 years	Risk of precocious closure of the epiphysis, severe skin damage (erythema multiforme, Stevens-Johnson syndrome, and toxic epidermal necrolysis), mental disorders, dyslipidemia, and benign intracranial hypertension. Caution in <12 years
PIM	Tretinoin	Oral tretinoin in children	Risk of leukocytosis, pseudo-brain tumor, and retinoic acid syndrome. Caution in children
PIM	Chlorhexidine	Chlorhexidine in neonates with very low birth weight (<1500 g)	Risk of chemical burns. Caution in neonates with very low birth weight (<1500 g)
**Antineoplastic And Immunomodulating Agents**			
PIM	L-Asparaginase	L-Asparaginase in children, especially in ≥10 years	Risk of leukoencephalopathy syndrome (RPLS), seizures, pancreatitis, coagulopathy, and abnormal blood glucose (children ≥10 years are more likely to happen). Caution in children (especially in ≥10 years)
PIM	Thalidomide	Cumulative doses >20 g or duration >10 months in Children	Risk of peripheral neuropathy. Caution in children. Cumulative doses >20 g or duration >10 months seem to increase the risk of peripheral neuropathy. It is recommended that children using thalidomide should be followed up every 3 months to identify and monitor possible side effects
PIM	Cyclosporine	Cyclosporine in <16 years, except for children receiving organ transplantation or with nephrotic syndrome	Risk of hirsutism, gingival hyperplasia, and nervous system damage. Avoid in <16 years, except for children receiving organ transplantation or with nephrotic syndrome
**Sensory Organs**			
PIM	Dexamethasone (ophthalmic)	Dexamethasone (ophthalmic) in children, especially in <10 years	Risk of high intraocular pressure and glaucoma. Caution in children (especially in <10 years)
PIM	Indomethacin	Indomethacin in <14 years	Risk of nephrotoxicity is higher than ibuprofen and acetaminophen/paracetamol. Caution in <14 years, and close monitoring of renal function during use is necessary
**Antiparasitic Products, Insecticides And Repellents**			
PIM	Lindane	Lindane in <10 years or <50 kg	Risk of seizures and convulsions. Avoid in <10 years or <50 kg
**Chinese Patent Medicine**			
PIM	Reduning injection	Reduning injection in children, especially in <2 years	Risk of severe allergic reactions such as anaphylactic shock, and dyspnea. Avoid in <2 years; Caution in children
PIM	Chuanhuning injection	Chuanhuning injection in children	Risk of severe allergic reactions such as anaphylactic shock, and dyspnea. Caution in children
PIM	Zedoray turmric oil injection	Zedoray turmric oil injection in children, especially in <10 years	Risk of severe allergic reactions such as anaphylactic shock, rash, and dyspnea. Caution in children (especially in <10 years). Special monitoring is required during use, and serious adverse reactions such as anaphylactic shock should be alerted
PIM	Qingkailing injection	Qingkailing injection in children, especially in <3 years	Risk of severe allergic reactions such as anaphylactic shock. Avoid in <3 years; Caution in children. Special monitoring is required during use, and serious adverse reactions such as anaphylactic shock should be alerted
PIM	Xiyanping injection	Xiyanping injection in children, especially in <2 years	Risk of severe allergic reactions such as anaphylactic shock. Avoid in <1 year; Caution in children (especially in <2 years). Special monitoring is required during use, and serious adverse reactions such as anaphylactic shock should be alerted
PIM	Asarone injection	Asarone injection in children, especially in <6 years or allergy-prone children	Risk of severe allergic reactions such as anaphylactic shock. Caution in children (especially in <6 years or allergy-prone children). Special monitoring is required during use, and serious adverse reactions such as anaphylactic shock should be alerted
PIM	Yanhuning injection	Yanhuning injection in children	Risk of severe allergic reactions such as anaphylactic shock. Caution in children. Special monitoring is required during use, and serious adverse reactions such as anaphylactic shock should be alerted
PIM	Houttuynia cordata injection	Houttuynia cordata injection in children	Risk of severe allergic reactions such as anaphylactic shock. Avoid in children. Special monitoring is required during use, and serious adverse reactions such as anaphylactic shock should be alerted
PIM	Tripterygium glycosides	Tripterygium glycosides in children, especially in boys	Reproductive toxicity (can still be found in boys who discontinued treatment for ≥6 months). Avoid in boys; Caution in girls. The risk of reproductive toxicity may increase when cumulative doses ≥200 mg/kg

Note: PIM, potentially inappropriate medication; PPO, potential prescribing omission; TDM, therapeutic drug monitoring; SSRIs: Selective serotonin reuptake inhibitors; SNRIs, Serotonin-norepinephrine reuptake inhibitors; AEDs, Antiepileptic drugs. Indications for each Chinese patent medicine: Reduning injection, upper respiratory infection (URI) and acute bronchitis; Chuanhuning injection, viral pneumonia and viral upper respiratory tract infection; Zedoray turmric oil injection, viral pneumonia, viral upper respiratory tract infection, peptic ulcer, viral hepatitis type A, viral enteritis, viral myocarditis and viral encephalitis; Qingkailing injection, acute hepatitis, upper respiratory infection (URI), pneumonia, cerebral thrombosis and cerebral hemorrhage; Xiyanping injection, bronchitis, amygdalitis, bacillary dysentery; Asarone injection, pneumonia, bronchial asthma, chronic obstructive pulmonary disease; Yanhuning injection, viral pneumonia, viral upper respiratory tract infection; Houttuynia cordata injection, pulmonary abscess, urinary tract infection, furuncle and carbuncle; Tripterygium glycosides: rheumatoid arthritis, dropsical nephritis, behcet disease, lepra reaction and autoimmune hepatitis.

The PIP criteria in children with specific diseases/conditions are mainly used to detect PIPs in children with a certain disease or condition, covering disease problems including respiratory problems (e.g., respiratory infections and asthma), neuropsychiatric disorders (e.g., nocturnal enuresis and attention deficit disorder with or without hyperactivity), dermatological problems (e.g., atopic eczema and acne vulgaris), digestive problems (e.g., gastroesophageal reflux and diarrhea), urinary problems (urinary infections), and other conditions (fever and pain). There were 17 propositions used to detect PIPs in children with respiratory diseases, which was the largest number (Table 2).

**TABLE 2:** PIP criteria for children with specific diseases/conditions.

PIM/PPO	Potentially inappropriate prescription	Risk/recommendation
**Respiratory problems**		
** Respiratory infections**		
PIM	An antibiotic for <4 days symptoms of acute upper respiratory tract infection (except: Bilateral acute otitis media in children younger than 2 years; Acute otitis media in children with otorrhoea; Acute sore throat/acute pharyngitis/acute tonsillitis when three or more CENTOR criteria are present; Significantly increasing CRP or the proportion of neutrophils; examinations of respiratory secretions suggesting bacterial infection)	In most cases, acute upper respiratory tract infection is self-limited, usually caused by the virus. Except for severe cases (anticipated to be no more than 20% of cases), do not need antibiotics. Abuse of antibiotics can induce antibiotic resistance
PIM	Antibiotics other than amoxicillin or penicillin V as the first-line treatment for acute otitis media, streptococcal pharyngitis, tonsillitis, or sinusitis, except for children allergic to amoxicillin and penicillin V	Compared with amoxicillin and penicillin V, other antibiotics have more adverse reactions and no better efficacy
PIM	Salicylates (including aspirin, methyl salicylate, magnesium salicylate, bismuth salicylate, magnesium choline trisalicylate, etc.) for children with suspected viral infection (flu and chickenpox)	Risk of Reye’s syndrome. Avoid in children with suspected viral infection (flu and chickenpox)
PIM	Corticosteroids for children with acute suppurative otitis media, nasopharyngitis, or streptococcal pharyngitis	No evidence that corticosteroids are effective and there are risks of adverse reactions
PIM	ICS for children with respiratory infections without chronic respiratory diseases	No evidence that ICS are effective and there are risks of adverse reactions
PIM	Nasal or oral decongestants (oxymetazoline, pseudoephedrine, naphazoline, ephedrine, phenylephrine) >7 days for children with acute upper respiratory tract infection	No definite evidence that decongestants are effective for acute upper respiratory tract infection complications (otitis media, sinusitis, etc.). Avoid >7 days when used to relieve symptoms such as nasal congestion
PIM	Sedating antihistamines (promethazine, chlorpheniramine, etc*.*) in <2 years, except for anaphylaxis	Risk of sedation. Weighing risks and benefits of use in children, avoid in <2 years
PPO	Acetaminophen/paracetamol combined with antibiotics to treat ear infections to relieve pain	Acetaminophen/paracetamol is more effective than placebo in reducing the 48-h pain of children with acute otitis media, and the incidence of adverse events is not significantly different from placebo
**Asthma**		
PIM	Ketotifen (or other antihistamines) for children with asthma	No definite curative effect
PPO	ICS should be prescribed to children 5–15 years old who are taking LABA	Maintenance treatment regimens recommended for children are all based on ICS, and other drugs are selectively used according to the condition of children
PPO	ICS should be prescribed to children ≥6 years old with asthma	Relief treatment: <6 years: As-needed SABA 6–11 years: As-needed low-dose ICS + SABA ≥12 years: As-needed low-dose ICS-formoterol (MART) (preferred) or as-needed low-dose ICS + SABA Maintenance treatment: Different doses of ICS ± LABA/LTRA (LABA is not recommended for children under 5 years old)
**Infantile bronchiolitis**		
PIM	Antibiotics or corticosteroids for children with bronchiolitis	No definite curative effect and risk of adverse reactions
PIM	H_1_-receptor antagonist, antitussive, mucolytics or ribavirin for children with bronchiolitis	No definite curative effect and risk of adverse reactions
**Cough**		
PIM	Mucolytics (acetylcysteine and carbocysteine) for children <2 years with acute cough associated with upper respiratory tract infection or acute bronchitis	No definite curative effect and safety in ＜2 years is unknown
PIM	Antibiotics for children with acute cough, except for children with significant signs of bacterial infection, general discomfort, or high-risk conditions (including severe comorbidities such as severe cardiac, pulmonary, renal, hepatic, or neuromuscular diseases, immunosuppression, or cystic fibrosis; premature neonates)	Acute cough, most commonly caused by viral upper respiratory tract infection or acute bronchitis, is generally self-limiting and resolves within 3–4 weeks without antibiotics. Abuse of antibiotics can induce antibiotic resistance
**Tuberculosis**		
PIM	Intravenous streptomycin instead of intramuscular streptomycin	Higher risk of respiratory muscle paralysis; Avoid intravenous streptomycin
**Tonsillectomy and adenoidectomy**		
PIM	Codeine for children after tonsillectomy and adenoidectomy	Risk of worsening dyspnea in children with pre-existing dyspnea after tonsillectomy and adenoidectomy. Avoid in children without CYP2D6 gene polymorphism testing after tonsillectomy and adenoidectomy
**Urinary problems**		
** Urinary infections**		
PIM	Antibiotics for children with asymptomatic bacterial urinary tract infection, except in the case of uropathy	Can induce antibiotic resistance
PIM	Antibiotic prophylaxis following an initial infection without complications, except in the case of uropathy	Can induce antibiotic resistance
**Dermatological problems**		
** Atopic eczema**		
PIM	Topical corticosteroids (medium and high titers) >14 days	The skin absorption rate of corticosteroids in children, especially infants, is higher than adults. Long-term (>14 days) use increases the hypothalamic-pituitary-adrenocortical axis (HPA) inhibition risk
PIM	High titers corticosteroids (0.05% clobetasol propionate, betamethasone dipropionate) are applied to the face, armpits, groin, or back of infants	The skin on the baby’s face, armpits, and groin is thin, so the absorption rate of topical corticosteroids is higher. Moreover, high titers corticosteroids (0.05% clobetasol propionate, betamethasone dipropionate) are more likely to cause the hypothalamus-pituitary-adrenal cortex axis (HPA) inhibition
PIM	Oral corticosteroids for children with atopic eczema	Unknown effect and many adverse reactions
PIM	Topical corticosteroids ≥ twice a day, except for severe lichenification	The effect of ≥twice a day does not increase and the risk of adverse reactions increases
PIM	Topical applied 0.03% tacrolimus ointment in children ≤2 years	Risk of skin burns. Not approved in children ≤2 years and not as mild as corticosteroids
PIM	Topical applied 0.1% tacrolimus ointment in children ≤16 years	Risk of skin burns. Not approved in children ≤16 years and not as mild as corticosteroids
** Acne vulgaris**		
PIM	Minocycline for children <8 years of age with acne	Risk of tooth discoloration, drug hypersensitivity syndrome (DHS), Stevens-Johnson syndrome, or lupus-like syndrome (LLS). Avoid in children <8 years with acne
PIM	Levonorgestrel, norgestrel, norethisterone, estradiol, dienogest, contraceptive implants, or vaginal rings are used to treat acne in children	Adverse effects on children’s growth, bone density, and thrombotic events
PPO	Oral or topical antibiotics should be used in combination with other drugs such as benzoyl peroxide (BP) and tretinoin	When used together with topical or systemic antibiotics, BP can reduce the incidence of antibiotic resistance in propionibacterium acnes and improve the effect
** Scabies**		
PIM	Benzyl benzoate for children with scabies	More irritating than permethrin or malathion, and no better effect
PPO	Ivermectin should be administered once a week after the first dose for children with scabies	A second dose of ivermectin a week later can kill scabies eggs and increase the effect
** Impetigo**		
PIM	Any antibiotic other than fusidic acid as the first-line treatment for children with impetigo, except for children allergic to fusidic acid	Other topical antibiotics do not have a better effect and are not as safe as fusidic acid
PIM	Combined use of topical and oral antibiotics	No evidence that the combination is better
** Herpes simplex**		
PIM	Topical corticosteroids for children with herpes simplex	May worsen the condition and prolong hospitalization
Ringworm		
PPO	Combination of topical and oral treatment	Ringworm requires systemic treatment because topical antifungal agents do not penetrate the hair follicles
**Digestive problems**		
** Nausea, vomiting, or gastroesophageal reflux**		
PIM	In the absence of feeding difficulties, pain, or growth retardation, acid inhibitors (PPI and H_2_-receptor antagonists) for children with gastroesophageal reflux, indigestion, crying without any other signs or symptoms, or syncope	No definite curative effect and risk of adverse reactions
PIM	Metoclopramide for children with nausea, vomiting, or gastroesophageal reflux	Risks of adverse reactions such as extrapyramidal reactions (dystonia and tardive dyskinesia) outweigh the benefits
PIM	Erythromycin for children with nausea, vomiting, or gastroesophageal reflux	No effective evidence in children with gastroesophageal reflux, and potential adverse reactions of nausea and vomiting, liver damage, allergic reactions, arrhythmias, and pyloric stenosis
PIM	Domperidone for children (especially <1 year) with nausea, vomiting, or gastroesophageal reflux	No effect in children with gastroesophageal reflux disease (GORD), and potential risk of serious cardiac and central nervous system (CNS) adverse reactions. For children <1 year whose blood-brain barrier is incomplete, the CNS adverse reactions risk is higher
PPO	Oral rehydration solution (ORS) for dehydrated children unless intravenous fluid therapy is indicated (shock, red flag symptoms despite ORS, persistent vomiting of ORS)	Children benefit significantly and risk is low
** Diarrhea**		
PIM	Loperamide for children <4 years or with acute infectious diarrhea	Loperamide has more risk than other diarrhea treatments, and there is no recommended dose for children <4 years
PIM	Antibiotics for children with diarrhea, except for the suspected or confirmed septicemia, the extra-intestinal spread of bacterial infection, <6 months with *salmonella* gastroenteritis, children who are malnourished or immunocompromised with *salmonella* gastroenteritis, *Clostridium* difficile-related pseudomembranous enterocolitis, Giardiasis, *Shigella* dysentery, Amoebic dysentery, cholera	Diarrhea is mostly caused by rotavirus and cryptosporidiosis. Misuse of antibiotics can cause unnecessary harm to patients and induce antibiotic resistance
PPO	Oral rehydration solution (ORS) for dehydrated children unless intravenous fluid therapy is indicated (shock, red flag symptoms despite ORS, persistent vomiting of ORS)	Children benefit significantly and risk is low
PPO	Intestinal microecological preparations (such as *Brucella*, Bifidobacterium, *Lactobacillus*, etc.) should be used for non-immunocompromised children with acute or antibiotic-related diarrhea to maintain the ecological balance of microorganisms in the intestinal tract	Children benefit significantly and risk is low
**Neuropsychiatric Disorders**		
** Nocturnal enuresis**		
PIM	Tricyclic antidepressants (such as desipramine, imipramine, etc.) as the first-line treatment for children with enuresis	Risk of sudden cardiac death outweighs the benefits in children with enuresis
PIM	Combined use of tricyclic antidepressants and anticholinergics for children with nocturnal enuresis	Combination use only increases the risk of adverse reactions rather than the effect
PIM	Desmopressin for children with only daytime symptoms	—
PIM	An anticholinergic agent used as a monotherapy in the absence of daytime symptoms	Addition of anticholinergics to children poorly controlled by desmopressin alone
** Attention deficit disorder with or without hyperactivity**		
PIM	Except for severe conditions, medications (such as amphetamine, methylphenidate) as first-line treatment in ≤6 years or using medications in ≤3 years	Behavioral interventions by parents or teachers are non-inferior to drug therapy and have no risk of adverse drug reactions
PIM	Two doses of sustained-release methylphenidate a day instead of just one	Due to its special pharmacokinetic characteristics, it only needs to be taken once a day, and twice a day will increase the cost and risk
PIM	Antipsychotics for children with attention deficit hyperactivity disorder	No significant benefit and risk of suicide
** Psychosis and schizophrenia**		
PIM	≥2 antipsychotic drugs are routinely prescribed for initial treatment	—
** Epilepsy**		
PIM	Carbamazepine, gabapentin, oxcarbazepine, phenytoin, pregabalin, or tiagabine for children with epileptic absence seizures	Exacerbate the condition, even induce a generalized seizure
PIM	Carbamazepine, gabapentin, oxcarbazepine, phenytoin, pregabalin, or tiagabine for children with myoclonic epilepsy	Exacerbate the condition, even induce a generalized seizure
PPO	TDM should be taken in children with uncontrolled seizures or obvious adverse reactions during medication, using multiple antiepileptic drugs, or having self-administered some unidentified medicines	TDM is recommended in children using antiepileptic drugs, which can clarify the absorption and distribution of drugs in the body and adjust doses by the individual situation to improve the effect and avoid or reduce potential adverse drug reactions
** Depression**		
PIM	SSRIs other than fluoxetine as first-line treatment (in the case of medication)	Fluoxetine is the only antidepressant with clinical trial evidence that its benefits outweigh the risks in children with depression
PIM	Tricyclic antidepressants (such as imipramine, amitriptyline, clomipramine, doxepin, etc.) for children with depression	Risk of sudden cardiac death outweighs the benefits
** Anorexia nervosa**		
PIM	Prescribing medication as the sole or primary treatment for children with anorexia nervosa	Compared with drugs, cognitive behavioral therapy has a significant effect and no risk of adverse drug reactions
Destructive or aggressive behaviors		
PIM	Antipsychotics for destructive and aggressive behaviors in children without autism, or emotional problems in children without bipolar disorder	Psychosocial interventions are effective and non-inferior to antipsychotics, and no risk of adverse drug reactions such as drowsiness, sedation, gastrointestinal discomfort, weight gain, etc.
**Other Conditions**		
** Fever**		
PIM	Alternate or combined use of two antipyretics (acetaminophen/paracetamol, ibuprofen) as first-line treatment	Alternate or combined use of two antipyretics is not more effective than monotherapy and long-term safety is unknown
PIM	Antipyretics (acetaminophen/paracetamol, ibuprofen) in children <2 months, < 38.2°C (axillary temperature), or without obvious discomfort	Physical cooling is recommended for children <2 months, < 38.2°C (axillary temperature), or without obvious discomfort
PIM	Ibuprofen or acetaminophen/paracetamol are used more than four doses per day, ibuprofen exceeds 40 mg/kg/d or acetaminophen exceeds 4 g/d	—
PIM	Corticosteroids as antipyretics for children with fever	—
PIM	Rectal rather than oral acetaminophen/paracetamol as first-line treatment for children with fever	—
** Pain**		
PIM	Drugs other than acetaminophen/paracetamol and ibuprofen as first-line pain relievers for children, except for migraine	Acetaminophen and ibuprofen are effective and safer for children than other painkillers
PIM	Opioids for children with migraine attacks	Risk of serious adverse reactions such as respiratory depression outweighs the benefits. Ibuprofen and acetaminophen/paracetamol (children and adolescents), and triptans (adolescents) are effective for children with migraine attacks

Note: PIM, potentially inappropriate medication; PPO, potential prescribing omission; TDM, therapeutic drug monitoring; SNRIs, Serotonin-norepinephrine reuptake inhibitors; AEDs, Antiepileptic drugs; ICS, inhaled corticosteroids; LABA, long-acting beta2 agonist; PPI, proton pump inhibitor.

## 4 Discussion

Pediatric patients are uniquely vulnerable to ADRs due to the immature organs and systems that metabolize and excrete drugs, and some medicines need to be used more cautiously in children (Kaushal et al., 2010; Davis, 2011). Davis’s study results showed that more than half of adverse drug events in pediatric inpatients were avoidable, and blocking medication errors at the prescribing stage might be one of the most effective preventive measures (Davis, 2011). It is increasingly recognized that rational prescribing is an important issue for children (Choonara, 2013). We developed a set of criteria for detecting PIPs in children through a modified Delphi method, with the target user population being healthcare workers who treat children under 18 years of age. The criteria can be used as a quality control tool for pediatric prescribing to improve medication safety in children. Moreover, the criteria can be used to investigate the prevalence of PIPs in children and track its changes over time to help evaluate the effectiveness of the implementation of relevant policies and measures.

### 4.1 Propositions with more controversy among panelists

There has been a heated debate among panelists as to whether “Nasal or oral decongestants for children with acute upper respiratory tract infection” should be included in the children’s PIP criteria. The Recommendation 1.3.3 in the “Sinusitis (acute): antimicrobial prescribing NICE guideline (NG79)” (Sinusitis, 2021) showed that “No evidence was found for using oral decongestants,” and the Recommendation 1.2.2 in the “Otitis media (acute): antimicrobial prescribing NICE guideline (NG91)” (NICE, 2021) also indicated that “Decongestants do not help symptom relief.” However, during the Delphi survey, some specialists commented that decongestants could relieve symptoms and improve the quality of life in children in time and the risk of short-term use might be small. Finally, considering the current evidence, specialists’ comments and actual clinical practice, we revised this proposition to “Nasal or oral decongestants >7 days for children with acute upper respiratory tract infection” and retained it.

No strong evidence was found to support the avoidance of fluoroquinolones in children. At present, there is no case report of severe and irreversible bone or cartilage damage in children caused by quinolones. Only the results of animal experiments showed that quinolones might permanently damage the soft tissues of the weight-bearing joints in young animals, causing the erosion of the weight-bearing joints or other joint diseases (Tatsumi et al., 1978; Gough et al., 1979). Moreover, in specific populations, such as children with complicated urinary tract infections, cystic fibrosis, and some community-acquired pneumonia cases, quinolones may have to be used (Jackson and Schutze, 2016). However, considering the Chinese National Children’s Formulary (Chinese National Formulary Editorial Committee, 2013) and the instructions of quinolones which clearly stated that “This product should be avoided in people under 18 years old,” this proposition was eventually retained in the children’s PIP criteria, with revising “Avoid in children” to “Caution in children.”

Aspirin is avoided in children with viral respiratory infections (flu and chickenpox) because of the risk of Raye’s syndrome. In the Delphi survey, some specialists questioned the authenticity of this association because the quality of the evidence was very low (Brunell et al., 1982; National surveillance for Reye syndrome, 1982; Surgeon General, 1982; Belay et al., 1999). However, this proposition was retained due to the serious harm of this adverse reaction (may lead to the death of children).

### 4.2 Comparison with existing children’s potentially inappropriate prescription criteria

The earliest PIP criteria for children is the POPI tool developed by French experts using the Delphi method (Prot-Labarthe et al., 2014)^,^ which is the basis for the POPI United Kingdom tool (Corrick et al., 2019) and the POPI Int tool (Sadozai et al., 2020) (both of them are formed after modifying the POPI tool). It consists of 105 PIP propositions that can be used by all medical professionals responsible for prescribing or dispensing medicines to children to detect potentially inappropriate medication and prescribing omission in pediatrics. Study results have shown the good applicability (Berthe-Aucejo et al., 2019a) and reliability (Berthe-Aucejo et al., 2019b) of this tool in French pediatric clinical practice. Thirty-one propositions in the POPI tool are consistent with our criteria (e.g., “Rectal rather than oral acetaminophen/paracetamol as first-line treatment for children with fever,” “Opioids for children with migraine attacks,” “Erythromycin as a prokinetic agent for children with nausea, vomiting or gastroesophageal reflux”, etc*.*), 24 propositions are different from our criteria (e.g., POPI: “Oral solutions of ibuprofen administered in more than three doses per day using a graduated pipette of 10 mg/kg (other than Advil),” “Loperamide before 3 years of age”; Our criteria: “Ibuprofen or acetaminophen/paracetamol are used more than four doses per day, ibuprofen exceeds 40 mg/kg/d or acetaminophen exceeds 4 g/d,” “Loperamide for children <4 years with acute infectious diarrhea,” etc*.*).

In 2020, after critical analysis, peer review, and public review, a list of drugs that are potentially inappropriate for use in pediatric patients has been developed and titled the “KIDs List” (Meyers et al., 2020), which contains 67 drugs and/or drug classes and 10 excipients. Twenty-three propositions in the list are consistent with our criteria [e.g., “Ceftriaxone; Kernicterus; Caution in neonates,” “Chloramphenicol; Gray baby syndrome; Avoid neonates, unless the blood concentration is monitored,” “Midazolam; Severe intraventricular hemorrhage, periventricular leukomalacia, or death; Avoid in neonates with very low birth weight (<1500 g),” etc.], 9 propositions are different from our criteria [e.g., KIDs List: “Azithromycin or erythromycin (oral and intravenous); Hypertrophic pyloric stenosis; Avoid in neonates,” “Valproic acid and derivatives; Pancreatitis, fatal hepatotoxicity; Avoid in infants, caution in <6 years”; Our criteria: “Azithromycin or erythromycin (oral or intravenous); Hypertrophic pyloric stenosis; Avoid in neonates ≤14 days,” “Valproic acid and its derivatives; Pancreatitis, fatal hepatotoxicity; Avoid in <2 years, especially in children with metabolic or mitochondrial diseases, or are taking other antiepileptic drugs such as phenytoin”].

### 4.3 Limitations

One of the main limitations of this study relates to use of the Delphi technique. Although it is a commonly used method, the reliability of the Delphi method for achieving consensus has been debated. The information gathered using a Delphi method represents only the views of chosen experts about a specific practice at a particular time and the results may vary depending on the experts included in the panel. In this study, to ensure the reliability of the final results, we invited 16 specialists with extensive pediatric clinical experience (All have been engaged in pediatric clinical work for more than 10 years, and more than half of them more than 20 years) to participate in a Delphi panel. Moreover, we also provided panelists with the best currently available evidence for each proposition during the Delphi process to help them better evaluate and comment. Second, this standard can only be used as a screening tool for potentially inappropriate prescriptions, and cannot directly determine the final rationality of prescriptions in place of comprehensive clinical assessment, especially in some patients with complex conditions. Under special situations, children using the drugs in children’s PIP criteria may be necessary after the children’s overall clinical situation has been fully assessed (prescriptions are appropriate at this time). Moreover, these criteria do not mandate absolute contraindications to any drug use in children and are only intended to provide medication warnings to pediatric clinicians or pharmacists. Third, only drugs that have been marketed in China and Chinese pediatric clinical practice were considered in the criteria forming process. Therefore, these PIP criteria may not directly apply to pediatric patients in other countries. However, other countries can modify the criteria based on the national drug listing situation and current clinical guidelines to improve its applicability. Finally, these criteria have not been tested in an actual clinical practice setting and remain to be validated. We will conduct two studies in the future. One study will measure the reliability of the criteria by examining the degree of consistency of PIP assessment results among users (Kappa), and the other study will evaluate the capacity of the criteria to detect PIPs in pediatrics to measure the clinical applicability and feasibility of the criteria.

### 4.4 Subsequent research and practice directions

Like the “STOPP/START criteria” (Huibers et al., 2019b) and the “PIM-Check criteria” (Blanc et al., 2018a; Blanc et al., 2018b) for the elderly, integrating our children’s PIP propositions into the clinical decision support system through computer coding algorithms to realize the automated identification and quantification of children’s PIP, which may be expected to improve the rationality of drug use in pediatric patients, reduce medication risk, and also contribute to the continuous improvement of medical quality.

## 5 Conclusion

A tool for screening potentially inappropriate prescriptions in children is formed to detect potentially inappropriate medication and prescribing omission in pediatrics and is available to all medical professionals liable to prescribe or dispense medicines to children. Moreover, we will conduct two subsequent studies to evaluate the reliability and clinical applicability of this tool.

## Data Availability

The original contributions presented in the study are included in the article/Supplementary Material, further inquiries can be directed to the corresponding author.

## Abbreviations

PIM, Potentially inappropriate medication; PIP, Potentially inappropriate prescription; PPO, Potential prescribing omission.
