# Supplementary material for: A tool for screening potentially inappropriate prescribing in Chinese children
Source: Front Pharmacol. 2022 Oct 31;13:1019795. doi: 10.3389/fphar.2022.1019795 (PMC9664213; doi:10.3389/fphar.2022.1019795)
Supplement: Supplementary file 1 [file DataSheet1.docx]

**Eligibility criteria**

**Inclusion criteria：**

1) PIP criteria or propositions, described as potentially dangerous or known ineffective prescription patterns (including drug contraindications, drug-drug interactions, drug-disease interactions, and high risks associated with inappropriate dose and route of administration, etc.), described as prescription patterns that do not conform to best clinical practices or current guidelines or described as prescription patterns that are easily ignored or omitted; 2) Target population: children (0-18 years).

**Exclusion criteria：**

1) No specific drug name or drug class was mentioned; 2) Related drugs are not marketed in China; 3) Potential risks to children caused by excipients in pharmaceutical prescriptions (such as benzyl alcohol, ethanol, etc.); 4) Duplicates; 5) Unobtainable full-texts; 6) Non-Chinese and non-English.

## Table Search strategy

| **Database** | **Medline (Ovid)** | **Embase (Ovid)** | **Cochrane library** |
| --- | --- | --- | --- |
| **Strategy** | 1 exp Pediatrics/ 60567  2 exp Adolescent/ 2109979  3 exp Child/ 1991198  4 exp Infant/ 1179127  5 1 or 2 or 3 or 4 3725757  6 exp Potentially Inappropriate Medication List/ 644  7 exp Inappropriate Prescribing/ 3815  8 (high risk medications or high risk prescriptions or omission prescriptions or PIPc or POPI).ab,kw,ti. 701  9 6 or 7 or 8 4712  10 5 and 9 675 | 1 exp Pediatrics/ 113358  2 exp Adolescent/ 1601125  3 exp Child/ 2762046  4 exp Infant/ 1031324  5 1 or 2 or 3 or 4 3592528  6 exp Potentially Inappropriate Medication List/ 1873  7 exp Inappropriate Prescribing/ 6503  8 (high risk medications or high risk prescriptions or omission prescriptions or PIPc or POPI).ab,kw,ti. 1429  9 6 or 7 or 8 7873  10 5 and 9 679 | ("Potentially Inappropriate Medication" or "potentially inappropriate drugs" or "Inappropriate Prescribing" or "high risk medications" or "high risk prescriptions" or "omission prescriptions" or "PIPc" or "POPI"):ti,ab,kw AND (Pediatrics or Adolescent or Child or Infant):ti,ab,kw |


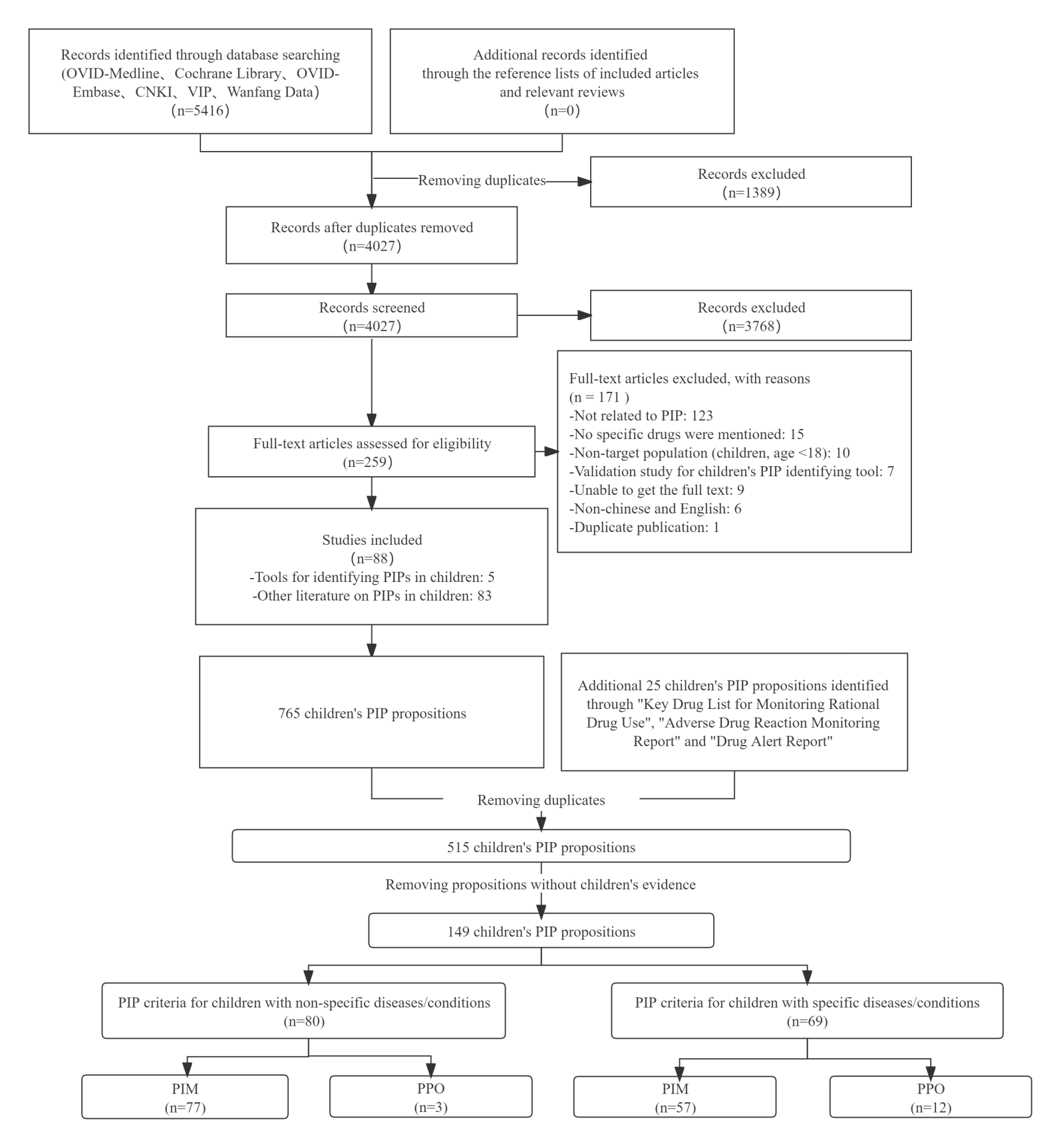


**Figure Results of literature and PIP propositions selection in the process of forming preliminary children’s PIP criteria.**

Note: PIP: Potentially inappropriate prescription; PIM: Potentially inappropriate medication; PPO: Potential prescribing omission.
